# Supplementary material for: Urinary renal epithelial cells can be used for NPHP1 phenotyping and a personalized therapeutic strategy
Source: J Cell Sci. 2025 Sep 8;138(20):jcs264141. doi: 10.1242/jcs.264141 (PMC12450468; doi:10.1242/jcs.264141)
Supplement: Supplementary information [file joces-138-264141-s1.pdf]

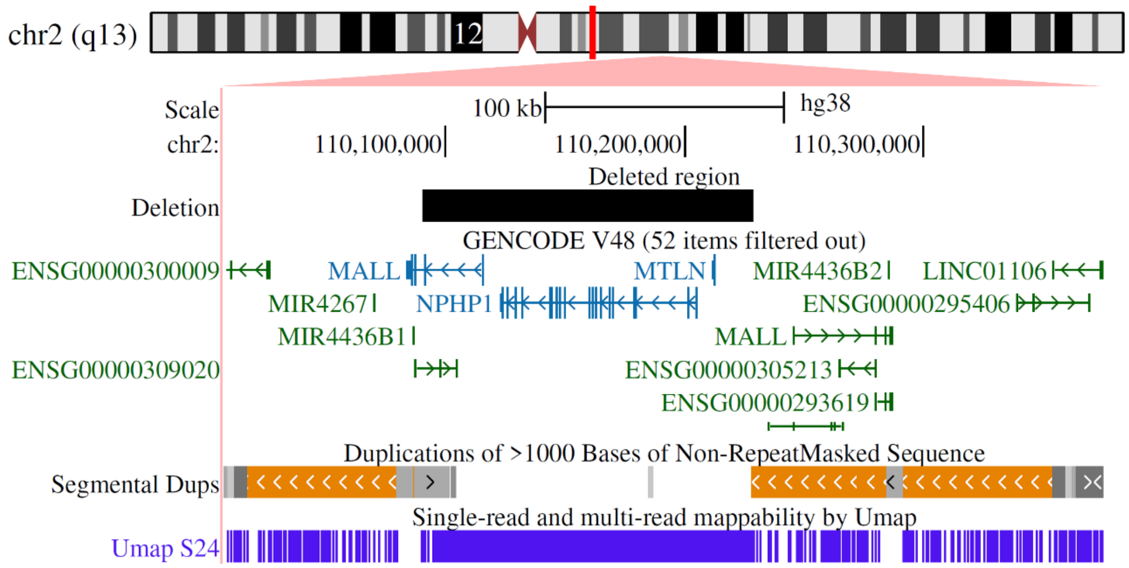

Fig. S1. Genomic context of the deletion. UCSC Genome Browser (hg38 assembly) view of the region on chromosome 2 encompassing the deleted segment identified in the proband and both parents. The custom track (black) indicates the genomic interval where BAM file alignments and read coverage support the presence of the deletion. Additional tracks shown include GENCODE v48 basic annotations, displaying protein-coding genes (blue) and non-coding genes (green), the Segmental Duplications track, and a mappability track indicating regions of high mapping quality in blue.

Journal of Cell Science • Supplementary information

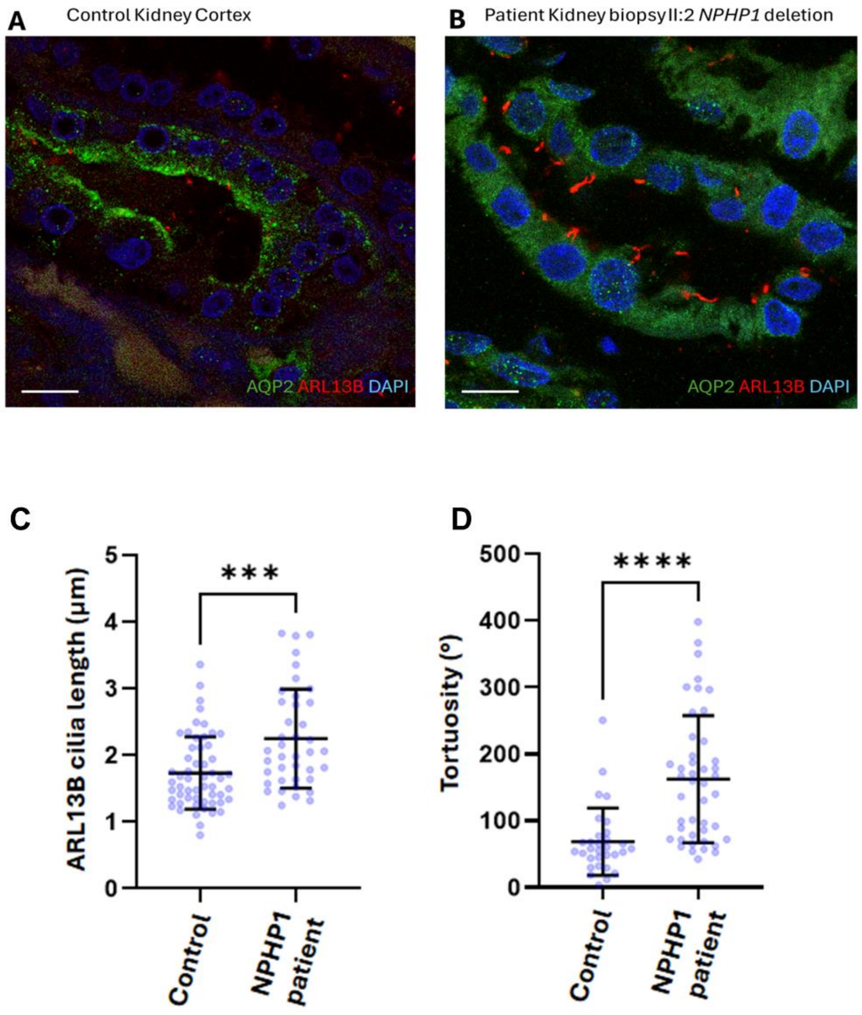

Fig. S2. Primary ciliary phenotypes in collecting ducts of human kidney biopsy tissue from *NPHP1* whole gene deletion patient

Renal biopsy tissue stained with DAPI (blue), aquaporin-2 (green) and ARL13B (red) in (A) healthy control and (B) patient II:2 with *NPHP1* whole gene deletion. Scale bar 10 µm. Quantification of primary cilia show that the (C) mean cilia length measured for the healthy control biopsy (n=55) was 1.73 µm compared to patient II:2 with *NPHP1* whole gene deletion (n=40) was 2.25 µm. Data shown as scatter plot with means. Error bar indicates mean with standard deviation *** p<0.0002, unpaired t test. Individual primary cilia tortuosity (D) was measured and plotted as sum of angels (°). The mean tortuosity of the healthy control (n=31) was 69° compared to the NPHP1 patient hURECs (n=44) was 162°. Error bar indicates mean with standard deviation *p<0.0001, unpaired t test.

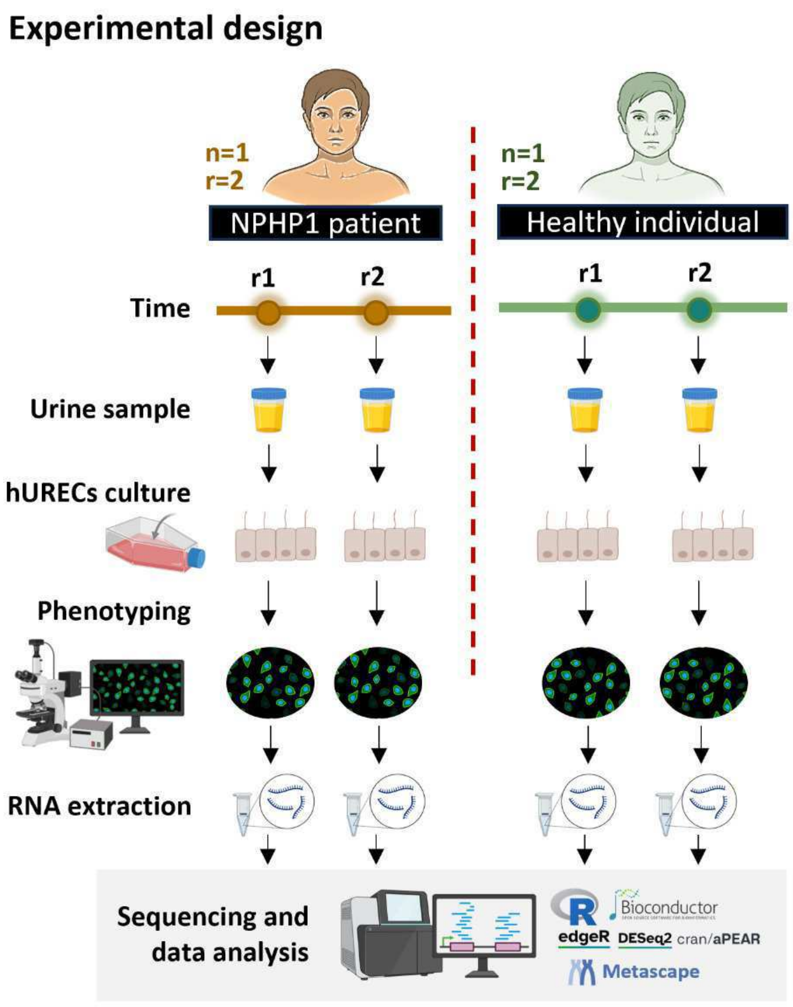

Fig. S3. Experimental design for deep phenotyping of cilia and transcriptomic analysis of hURECs from (II:3) and a healthy individual.

The study involves one NPHP1 patient (n = 1) and one healthy individual (n = 1), each sampled in duplicate (r = 2). Urine samples were collected at two different time points (r1 and r2) for each subject hURECs were cultured from the urine samples, followed by immunofluorescence imaging and RNA extraction. Percentage of ciliated cells, as well as mean cilia length and tortuosity, were quantified. Extracted RNA underwent sequencing, and the data were analysed using the edgeR and DESeq2 R packages, as well as Metascape.

Journal of Cell Science • Supplementary information

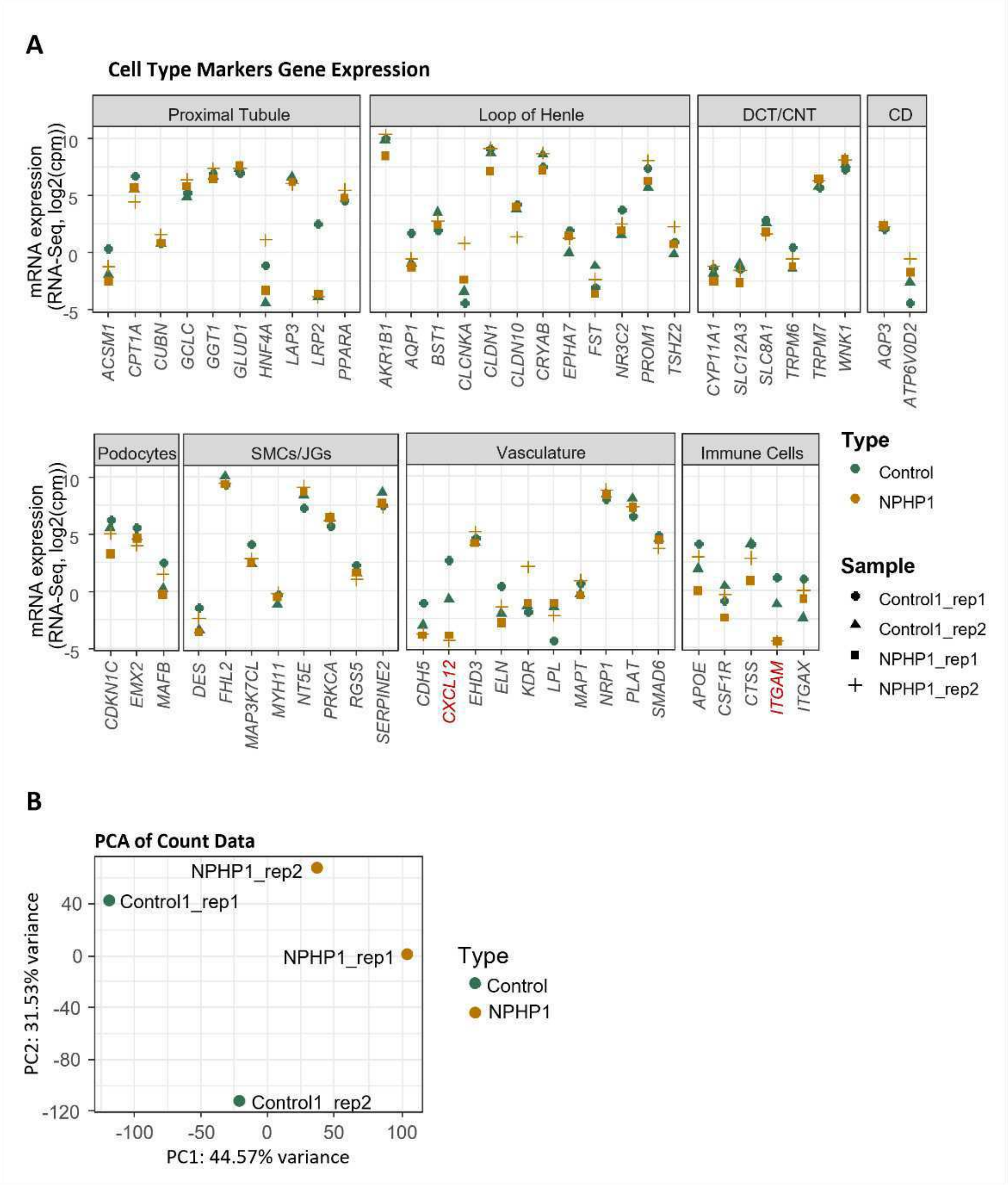

Fig. S4. Expression of cell type gene markers and PCA plot from (II:3) and control hURECs.

(A) Expression levels of cell type marker genes across samples. CPM (counts per million) values for selected genes that serve as markers for specific cell types from different parts of the nephron (Balzer, Rohacs et al. 2022) (Garcia, Serafin et al. 2022) were plotted. Genes shown in red are differentially expressed genes. **(B)** Principal Component Analysis (PCA) plot of gene expression data (log2 CPM).

Journal of Cell Science • Supplementary information

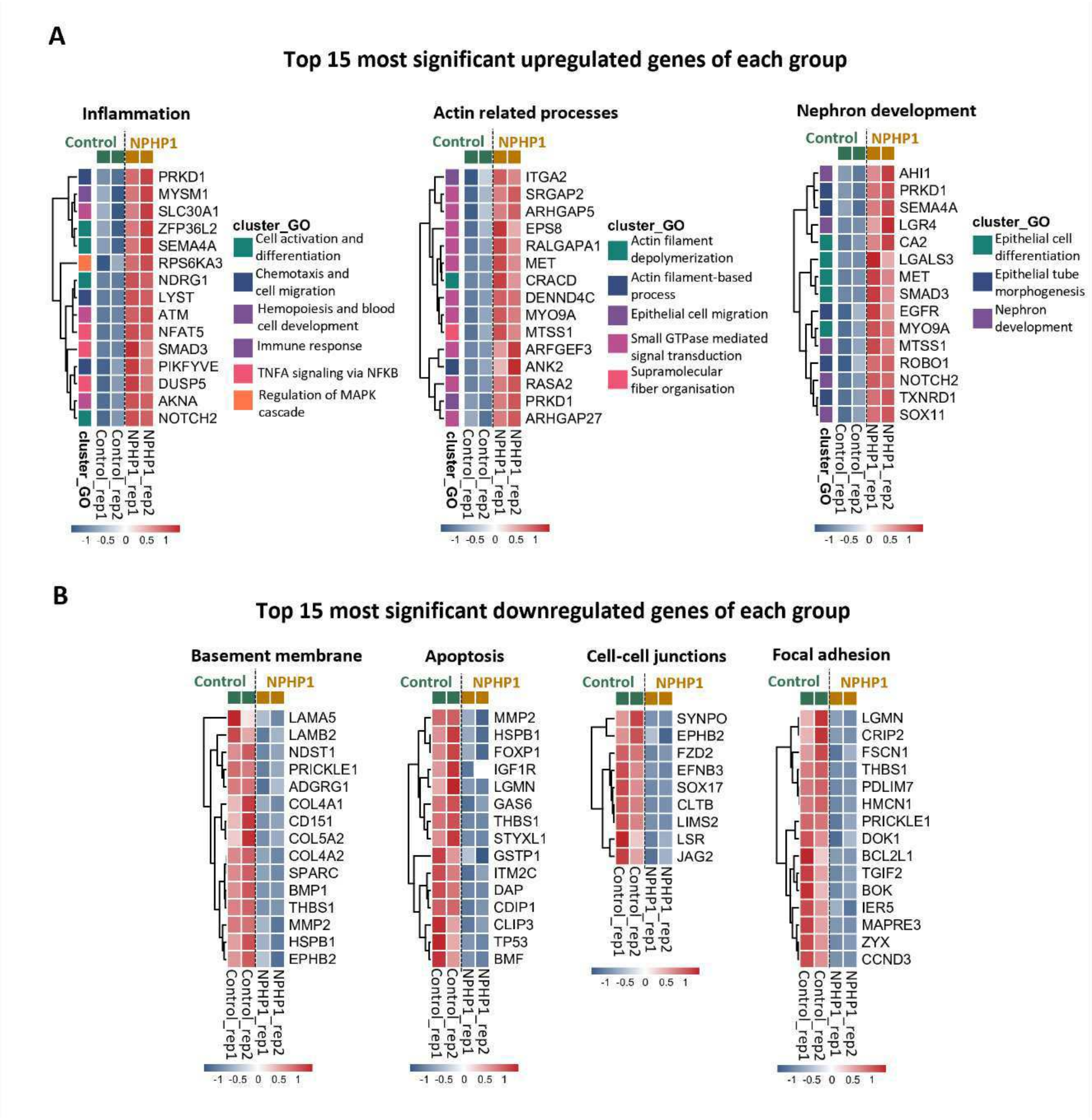

Fig. S5. Heatmaps of the top significantly dysregulated genes across relevant pathways.

(A) Heatmap of the top 15 upregulated genes with the lowest standard error of log fold change. **(B)** Heatmap of the top 15 downregulated genes with the lowest standard error of log fold change. For both panels, the genes displayed are selected from distinct, biologically relevant categories identified through pathway enrichment analysis.

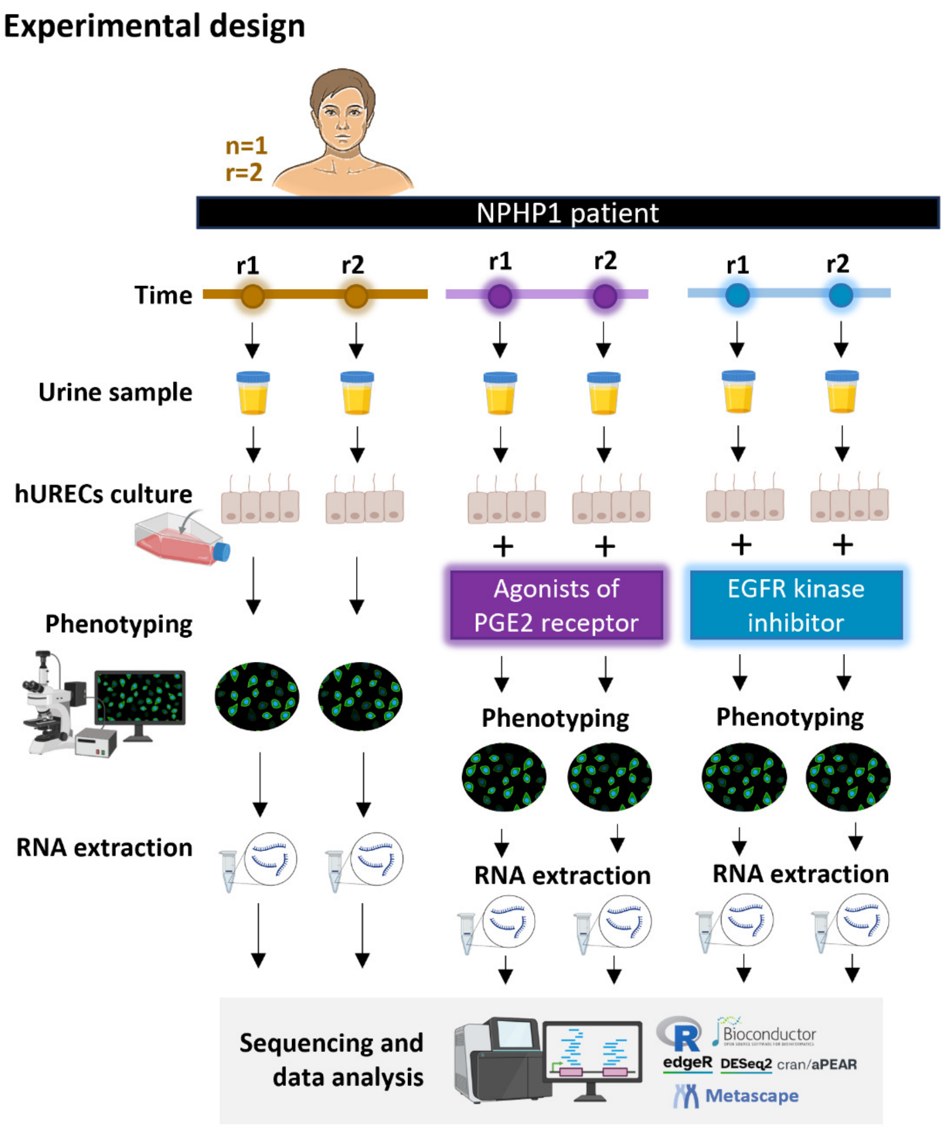

Fig. S6. Experimental workflow for hUREC treatment with ALP and an EGFR kinase inhibitor.

Urine samples were collected at two time points (r1 and r2). hURECs were cultured at each time point. The cultured cells underwent passaging and splitting for expansion. hURECs were then cryopreserved (frozen) and later revived for experimental treatments. Revived cells were treated with ALP, an agonists of the PGE2 receptor and an EGFR kinase inhibitor to assess their effects in the ciliary and transcriptional phenotypes.

Journal of Cell Science • Supplementary information

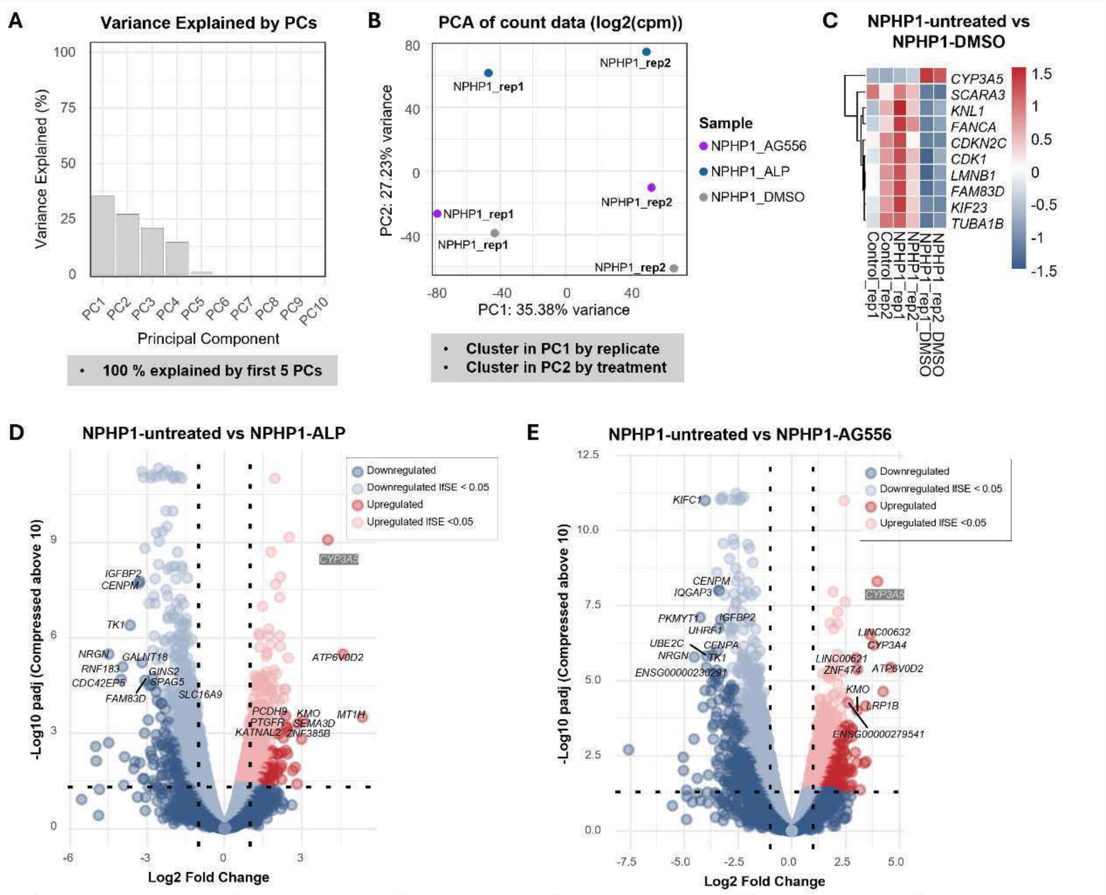

Expression Status	Num. Genes
Upregulated	1637
Downregulated	1837
Not significant	7956

Expression Status	Num. Genes
Upregulated	1638
Downregulated	2124
Not significant	11357

Fig. S7. Principal component and differential gene expression analyses in treated II:3 hURECs.

(A) Bar plot showing the variance explained by the first 10 principal components (PCs) in the PCA of treated (II:3) hURECs, with 100% of the variance explained by the first five PCs. **(B)** Principal Component Analysis (PCA) plot of batch-corrected gene expression data (log_2 CPM). Samples cluster by replicate in PC1 and by treatment in PC2. **(C)** Heatmap of significantly differentially expressed genes (DEGs) (padj < 0.05, $|log_2(FC)| > 0.58$) between untreated patient hURECs and patient hURECs treated with DMSO. **(C-D)** Volcano plots and tables showing dysregulated genes identified by DESeq2 analysis for **(C)** untreated patient hURECs vs. AG556-treated patient hURECs and **(D)** untreated patient hURECs vs. ALP-treated patient hURECs**.** The top 10 genes are labelled, and genes with a grey background are also dysregulated in the comparison between DMSO-treated and untreated patient hURECs.

Journal of Cell Science • Supplementary information

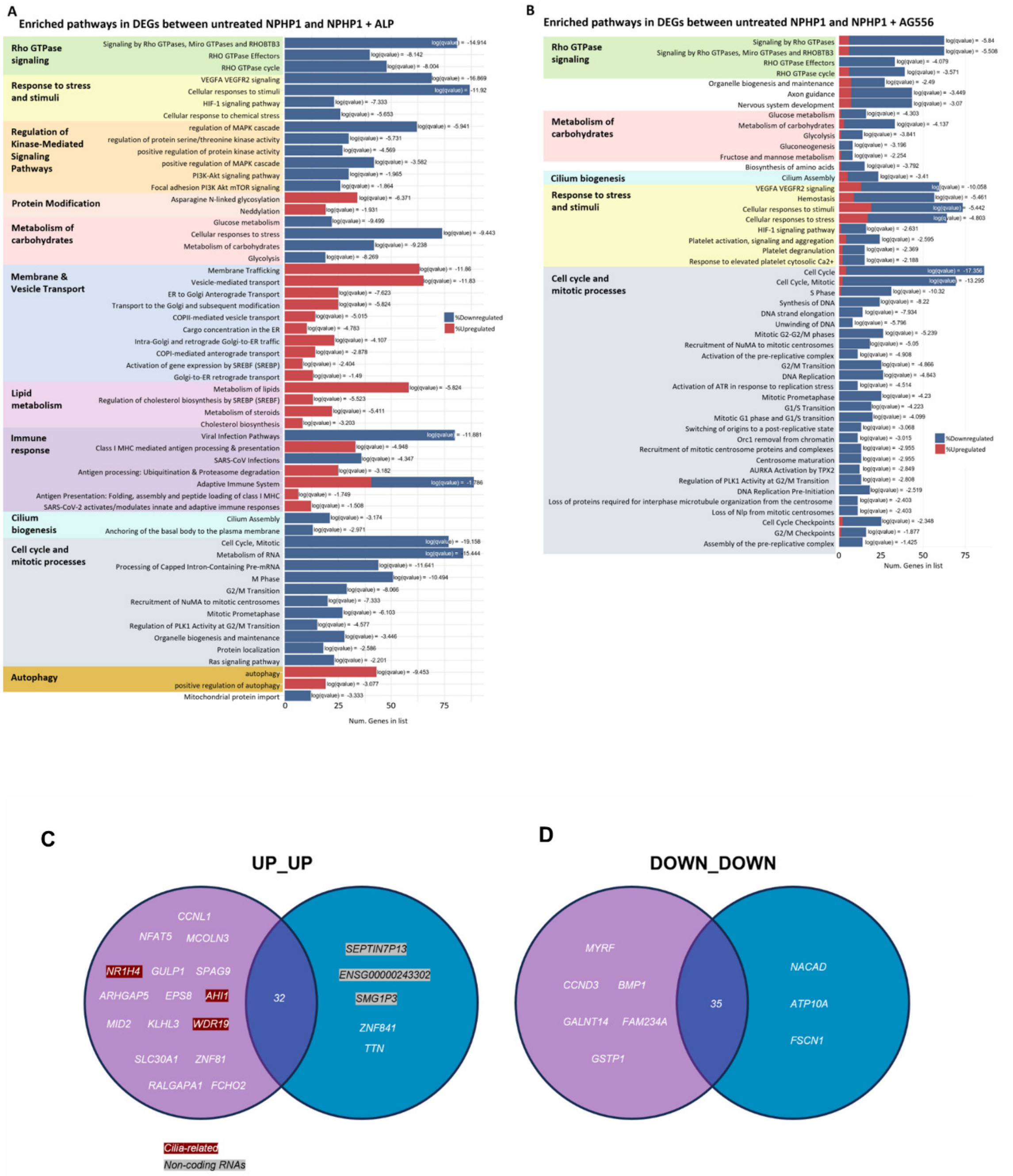

Fig. S8. Dysregulated pathways in treated patient hURECs and the effects of treatments on enhancing the disease signature.

Bar plots showing Metascape pathway enrichment analysis results for Reactome pathways and non-redundant pathways from WikiPathways or KEGG databases. Only significant pathways (padj < 0.05) are included. **(A)** Enriched pathways in differentially expressed genes between untreated patient hURECs and AG556-treated patient hURECs. **(B)** Enriched pathways in

Journal of Cell Science • Supplementary information

differentially expressed genes between untreated patient hURECs and ALP-treated patient hURECs. Venn diagrams illustrating the genes that are upregulated **(C)** or downregulated **(D)** in both treatments (ALP and AG556). This analysis is based on the UP_UP and DOWN_DOWN gene sets described in Figure 8A. The intersections highlight the number of genes that are similarly affected by both treatments, indicating a shared impact on the disease signature (see Table S9 for the full list). Genes with a red background are cilia-related genes from the SYSCILIA database (Vasquez et al., 2021), while genes with a grey background are non-protein-coding genes.

Table S1. Differential gene expression analysis results from DESeq2 comparing untreated patient II:1 hURECs to control hURECs. Each row represents a gene annotated by its Ensembl ID. Columns include: baseMean (average normalised expression across all samples), log2FoldChange (log2-transformed fold change between patient and control), lfcSE (standard error of the log2 fold change), stat (Wald test statistic), pvalue (raw p-value), and padj (adjusted p-value using the Benjamini-Hochberg method). Upregulated genes are highlighted in blue and Downregulated genes in red.

Available for download at
https://journals.biologists.com/jcs/article-lookup/doi/10.1242/jcs.264141#supplementary-data

Table S2. Primer sequences used in qPCR validation of differentially expressed genes between untreated patient II:1 hURECs and control hURECs.

Available for download at
https://journals.biologists.com/jcs/article-lookup/doi/10.1242/jcs.264141#supplementary-data

Table S3. Metascape pathway enrichment analysis of differentially expressed genes between untreated patient II:1 hURECs and control hURECs. Each row corresponds to a significantly enriched pathway or biological process. Columns include: Category, the source database used (e.g., GO, KEGG, Reactome); Term: the identifier for the pathway in the database; Description: a brief summary of the pathway/process; LogP and Log(q-value): the log-transformed p-value and adjusted p-value for enrichment significance, respectively; Genes and Symbols: the input gene Entrez Ids. and their corresponding gene symbols involved in the enriched term; InTerm_InList: ratio of input genes found in the enriched term; Dysregulation type: whether the input genes were upregulated or downregulated in the patient relative to control.

Available for download at
https://journals.biologists.com/jcs/article-lookup/doi/10.1242/jcs.264141#supplementary-data

Table S4. Differential gene expression analysis results from DESeq2 comparing untreated patient II:1 hURECs to DMSO-treated patient II:1 hURECs . Each row represents a gene annotated by its Ensembl ID. Columns include: baseMean (average normalized expression across all samples), log2FoldChange (log2-transformed fold change between patient and control), lfcSE (standard error of the log2 fold change), stat (Wald test statistic), pvalue (raw p-value), and padj (adjusted p-value using the Benjamini-Hochberg method). Upregulated genes are highlithed in blue and Downregulated genes in red.

Available for download at
https://journals.biologists.com/jcs/article-lookup/doi/10.1242/jcs.264141#supplementary-data

Table S5. Differential gene expression analysis results from DESeq2 comparing untreated patient II:1 hURECs to ALP-treated patient II:1 hURECs . Each row represents a gene annotated by its Ensembl ID. Columns include: baseMean (average normalized expression across all samples), log2FoldChange (log2-transformed fold change between patient and control), lfcSE (standard error of the log2 fold change), stat (Wald test statistic), pvalue (raw p-value), and padj (adjusted p-value using the Benjamini-Hochberg method). Upregulated genes are highlithed in blue and Downregulated genes in red.

Available for download at
https://journals.biologists.com/jcs/article-lookup/doi/10.1242/jcs.264141#supplementary-data

Table S6. Differential gene expression analysis results from DESeq2 comparing untreated patient II:1 hURECs to AG556-treated patient II:1 hURECs . Each row represents a gene annotated by its Ensembl ID. Columns include: baseMean (average normalized expression across all samples), log2FoldChange (log2-transformed fold change between patient and control), lfcSE (standard error of the log2 fold change), stat (Wald test statistic), pvalue (raw p-value), and padj (adjusted p-value using the Benjamini-Hochberg method). Upregulated genes are highlithed in blue and Downregulated genes in red.

Available for download at
https://journals.biologists.com/jcs/article-lookup/doi/10.1242/jcs.264141#supplementary-data

Journal of Cell Science • Supplementary information

Table S7. Metascape pathway enrichment analysis of differentially expressed genes between untreated patient II:1 hURECs and ALP-treated patient II:1 hURECs. Each row corresponds to a significantly enriched pathway or biological process. Columns include: Category, the source database used (e.g., GO, KEGG, Reactome); Term: the identifier for the pathway in the database; Description: a summary of the pathway/process; LogP and Log(q-value): the log-transformed p-value and adjusted p-value for enrichment significance, respectively; Genes and Symbols: the input gene Entrez Ids. and their corresponding gene symbols involved in the enriched term; InTerm_InList: ratio of input genes found in the enriched term; Dysregulation type: whether the input genes were upregulated or downregulated in the untreated relative to treated hURECs.

Available for download at
https://journals.biologists.com/jcs/article-lookup/doi/10.1242/jcs.264141#supplementary-data

Table S8. Metascape pathway enrichment analysis of differentially expressed genes between untreated patient II:1 hURECs and AG556-treated patient II:1 hURECs. Each row corresponds to a significantly enriched pathway or biological process. Columns include: Category, the source database used (e.g., GO, KEGG, Reactome); Term: the identifier for the pathway in the database; Description: a summary of the pathway/process; LogP and Log(q-value): the log-transformed p-value and adjusted p-value for enrichment significance, respectively; Genes and Symbols: the input gene Entrez Ids. and their corresponding gene symbols involved in the enriched term; InTerm_InList: ratio of input genes found in the enriched term; Dysregulation type: whether the input genes were upregulated or downregulated in the untreated relative to treated hURECs.

Available for download at
https://journals.biologists.com/jcs/article-lookup/doi/10.1242/jcs.264141#supplementary-data

Table S9. Overlap of differentially expressed genes between untreated patient II:1 hURECs vs control hURECs (disease signature) and untreated vs treated patient hURECs (treatment effect). This table supports the transcriptional signature interpretation presented in Figure 8.A, highlighting genes with rescued or enhanced expression patterns following treatment. Each row corresponds to a gene annotated by its Ensembl ID, gene name, and Entrez ID. Columns labelled .x refer to the untreated patient vs control hURECs comparison, and columns labelled .y refer to the untreated vs treated patient hURECs comparison. These include: baseMean, log2FoldChange, lfcSE, stat, pvalue, and padj for each comparison, opposite.FC.Sign: indicates whether the gene shows an opposite direction of change (rescue) or same direction (enhancement) between comparisons; comparison:XvsY: identifier for the comparison pair.

Available for download at
https://journals.biologists.com/jcs/article-lookup/doi/10.1242/jcs.264141#supplementary-data

Table S10. Pathway enrichment analysis of genes similarly regulated by both ALP and AG556 treatments. This table presents the results of pathway enrichment analysis using genes that are upregulated (UP_UP) or downregulated (DOWN_DOWN) in both treatments. Each row corresponds to a significantly enriched biological process or pathway, sourced from databases such as GO, KEGG, or Reactome. Gene set: indicates if the gene group was upregulated or downregulated in both treatments.

Available for download at
https://journals.biologists.com/jcs/article-lookup/doi/10.1242/jcs.264141#supplementary-data

Table S11. Differentially expressed ciliary genes (from SYSCILIA and CiliaCarta databases) across multiple comparisons involving II:1 patient hURECs. Each row represents a gene annotated by Ensembl ID and gene name. Columns 3 to 6 indicate whether the gene is significantly upregulated (UP), downregulated (DOWN), or not significantly changed (FALSE) in the following comparisons: untNPHP1_vs_Control: untreated patient vs control hURECs (disease signature); untNPHP1_vs_NPHP1-ALP: untreated vs ALP-treated patient hURECs; untNPHP1_vs_NPHP1-AG556: untreated vs AG556-treated patient hURECs; untNPHP1_vs_NPHP1-DMSO: untreated vs DMSO-treated patient hURECs (vehicle control).

Available for download at
https://journals.biologists.com/jcs/article-lookup/doi/10.1242/jcs.264141#supplementary-data
